# Effects of High Consumption of Vegetables on Clinical, Immunological, and Antioxidant Markers in Subjects at Risk of Cardiovascular Diseases

**DOI:** 10.1155/2018/5417165

**Published:** 2018-10-08

**Authors:** Ilaria Peluso, Anna Raguzzini, Giovina Catasta, Vittoria Cammisotto, Anna Perrone, Carlo Tomino, Elisabetta Toti, Mauro Serafini

**Affiliations:** ^1^Research Centre for Food and Nutrition, Council for Agricultural Research and Economics (CREA-AN), Rome, Italy; ^2^Research and Development, IRCCS San Raffaele Pisana, Rome, Italy; ^3^Functional Food and Metabolic Stress Prevention Laboratory, Faculty of Biosciences and Technologies for Agriculture, Food and Environment, University of Teramo, Teramo, Italy

## Abstract

High intakes of vegetables have been associated with a lower incidence of cardiovascular diseases (CVD). However, the effect of vegetables on immune function and antioxidant status in human studies have provided contrasting results. In the present study, after a week of run-in period, 38 subjects at risk of CVD were randomly assigned to one of the following 4-week interventions: low vegetable consumption (800 g of vegetables/week) or high vegetable consumption (4200 g of vegetables/week). Vegetables included carrots, topinambur (Jerusalem artichoke, *Helianthus tuberosus*), tomatoes, red cabbage, and sweet peppers. Blood and salivary samples were collected before and after intervention periods. In addition to clinical, immunological, and antioxidant markers, leukocyte and lymphocyte expression of the gut-homing *β*7 integrin was evaluated. No significant changes were detected in clinical, immunological, and antioxidant markers in biological samples, except for an increase in white blood cell count for the low vegetable consumption group (*p* < 0.05). The study provides additional evidence about the uncertainty of providing a clear evidence for vegetables in modulating markers of immune function and antioxidant status. Further studies are needed in order to unravel the mechanism of effect of vegetable consumption in cardiovascular prevention.

## 1. Introduction

High intakes of fruits and vegetables have been associated with a lower incidence of cardiovascular diseases (CVD), due to their antioxidant and anti-inflammatory properties [1]. Data from a meta-analysis reported that the plant-derived food category, such as chocolate, fruits, and vegetables, showed a clear antioxidant response after acute ingestion, whereas only vegetables were able to increase plasma antioxidant capacity after chronic intervention trials [2]. However, some studies did not find improvement of oxidative DNA damage [3, 4] and lipid peroxidation markers [4] after the consumption of 600–800 g/d of fruits and vegetables for 24/28 days in healthy nonsmokers. In healthy subjects, results are conflicting when both antioxidant and inflammatory markers were measured in the same study after consumption of carrot juice [5], tomato juice [6], and Lyc-o-Mato in the form of drink [7] or supplement [8, 9]. In healthy nonsmoking men, 8 servings/d of carotenoid-rich vegetables and fruit for 4 wk did not change immunologic markers, including the number and activity of natural killer cells, secretion of cytokines, and lymphocyte proliferation [10] However, it must be taken into account that some of the intervention studies with vegetable-derived products (including juices and extracts) were not controlled for placebo and/or were conducted on healthy subjects [11]. In this context, it must be taken into account that both antioxidant [2] and anti-inflammatory [12] effects are more evident in subjects with CVD risk factors when compared with healthy subjects [2]. Therefore, the aim of this study was to evaluate the effects of high consumption of vegetables in subjects with risk factors for CVD on clinical, immunological, and antioxidant markers. For the intervention study, we selected topinambur for the previous observed improvement of glucose metabolism [13] and tomato, red cabbage, and/or carrot for the lipid lowering, anti-hypertensive, anti-coagulant, antioxidant, anti-inflammatory, and immunomodulating activities [14–22]. Immunomodulation has been reported *in vitro* also for pectin from sweet pepper [23], whereas red cabbage anthocyanins have been proposed as inhibitors of lipopolysaccharide-induced oxidative stress in blood platelets, indirectly by their antioxidant properties and directly by binding with Toll-like receptors (TLR) [24]. From that, in addition to commonly used clinical, immunological, and antioxidant markers, for the first time, we evaluated the effect of high vegetable consumption on the leukocyte and lymphocyte expression of the gut-homing *β*7 integrin, being a clinical relevant target for treatment [25].

## 2. Materials and Methods

### 2.1. Recruitment and Selection of the Subjects

Italian subjects were recruited by advertisements. Adult individuals (*n* = 60, 30 men and 30 women) aged between 26 and 65 years were selected (for women, a proven absence of menopause was required, being postmenopausal status an independent risk factors for CVD [26]) on the basis of the following criteria. Exclusion criteria: any pathology (including allergies and gastrointestinal disorders that reduce or alter nutrient absorption), use of drugs or supplements and special diet regimens (vegetarian or vegan). Inclusion criteria: at least one of the following risk factors for CVD: waist circumference > 102 cm (men) and 88 cm (women) and/or waist/hip circumference ratio > 0.95 (men) and 0.8 (women); total cholesterol > 200 mg/dl and/or HDL < 35 (men) and 40 (women) mg/dl and/or triglycerides > 150 mg/dl; smoking habit; sedentary lifestyle; and low fruit and vegetable consumption (maximum 4 portions/week).

**Figure 1:**
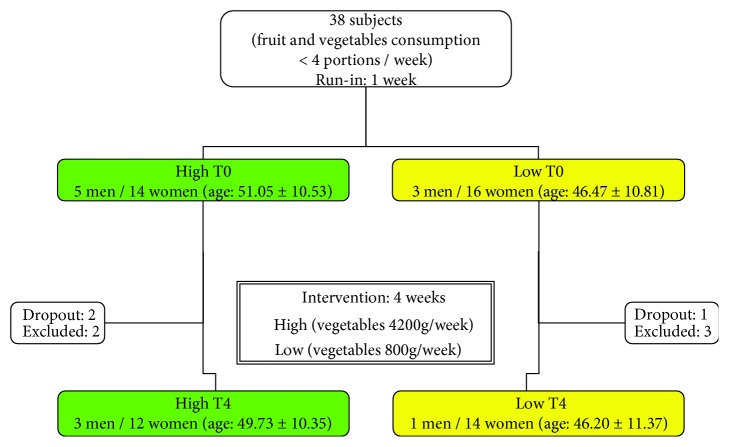
Flow chart of study.

Frequency of fruit and vegetable intake assessed by the validated 14-item questionnaire [27] and physical activity was evaluated according to the “Guidelines for Data Processing and Analysis of the International Physical Activity Questionnaire” (IPAQ) [28, 29]. Of the 60 selected subjects with a low consumption of fruit and vegetables, 38 subjects agreed to participate in the study and were recruited (Figure 1), 3 subjects left the study for personal reasons (1 of group low and 2 of group high), and 5 subjects (3 group low and 2 group high) reported symptoms of viral influence during the study; for this reason, they have been excluded. At the end of the follow-up, 30 compliant subjects (15 for each group) were included (Figure 1). All subjects had low physical activity level and had a low consumption of fruit and vegetables at baseline. Percentage of smokers and dyslipidaemic subjects and macronutrient intakes in the two groups after randomization are described in Table 1.

### 2.2. Vegetables

In order to standardize the vegetables consumed between subjects, carrots, topinambur, tomatoes, red cabbage, and sweet peppers were purchased by an organic farming company and were delivered weekly (Biobox S.r.l., Italy) to subjects' homes. During the study period, 4 different samples of vegetables were collected and analysed in triplicate for their antioxidant and phenolic content. Extracts containing the medium polar compounds (ANFI-Ex) and the water extracts (W-Ex) of vegetables were obtained as previously described [30]. The ferric-reducing antioxidant power (FRAP) assay was utilized to measure the antioxidant capacity of vegetables and the total phenol content (TPC) was measured using the Folin Ciocalteu assay, as previously described [31].

### 2.3. Study Design and Intervention

After a week of run-in period (Figure 1), in a parallel design, the subjects (*n* = 38) were randomly assigned to one of the following 4-week interventions:

- Low (control, *n* = 19) vegetable consumption: ≈800 g of vegetables/week (152 g carrots, 152 g topinambur, 152 g tomatoes, 152 g red cabbage, and 190 g sweet peppers)
- High (increased consumption of vegetables, *n* = 19): 4200 g of vegetables/week (800 g carrots, 800 g topinambur, 800 g tomatoes, 800 g red cabbage, and 1000 g sweet peppers). Compliance to dietary instructions was evaluated by 3-day food diaries. During the 4-week intervention, apart from the consumption of vegetables, subjects did not change dietary and lifestyle habits and at the end of the intervention intrasubject BMI did not change compared to baseline level

### 2.4. Collection and Analysis of Samples

On subjects fasting for at least 12 hours, venous blood samples were taken, according to the good clinical practice at Ospedale San Raffaele Pisana, Rome, and samples of saliva (Salivette, Sarstedt) were collected.

Clinical markers (glucose, insulin, total cholesterol: TC, low-density lipoproteins: LDL, high density lipoproteins: HDL, triglycerides: TG, proteins, uric acid: UA, and direct, indirect, and total bilirubin: dir-BR, ind-BR, and BR), plasma immunoglobulins, markers of thrombotic risk (fibrinogen and D-dimer), and complete blood count, including mean platelet volume (MPV) and platelet distribution width (PDW), were measured according to the good clinical practice at Ospedale San Raffaele Pisana, Rome [32–34].

Salivary UA levels were measured by colorimetric kits provided by Sentinel CH. SpA. HOMA-IR was calculated from glucose and insulin values. Salivary immunoglobulins A (IgAs) (LS bio), plasma transforming growth factor- (TGF-) *β* (RayBiotech) and interleukin- (IL-) 17 (RaybBiotech) were measured with ELISA kits. Plasma IL-2, IL-4, IL-6, IL-10, interferon- (INF-) *γ*, and tumor necrosis factor- (TNF-) *α* were measured with CBA kit (BD).

Immunophenotype was analysed on a Coulter Epics XL-MCL (Beckman Coulter), using monoclonal antibodies labelled with four fluorescent dyes, fluorescein isothiocyanate (FITC: CD16by R&D systems and CD45RA andCD127 by BD), phycoerythrin (PE: CD8, CD19, and CD25 all by R&D systems), phycoerythrin-Texas Red (ECD: CD3, CD4, and CD14 all by Beckman Coulter), and PhycoerythrinCyanin 5 (PC5: integrin *β*-7 by BD). Forward scatter (FSC) and side scatter (SSC) were acquired on a linear scale and fluorescence was acquired on a logarithmic scale. The percentage of leukocytes expressing the gut homing *β*-7 integrin was evaluated in the gated regions of granulocytes (FSC^low^SSC^high^), monocytes (CD14^+^CD4^dim^), natural killer cells (NK, CD3^−^CD8^dim^CD16^+^), B lymphocytes (CD19^+^), T lymphocytes (CD3^+^), T-cytotoxic lymphocytes (CD3^+^CD8^+^), T-helpers (CD4^+^), naïve (CD45RA^+^) and memory (CD45RA^−^) T cells, and T-helpers with different expression of IL-2 receptor (CD25^−^, CD25^dim^, CD25^bright^) including regulatory T cells (Treg CD4^+^CD25^bright^CD127^−^).

For platelet-rich plasma (PRP), blood was collected in citrate tubes (BD Vacutainer), centrifuged at 180 *g* for 15 minutes, and washed platelets were obtained as previously described [35]. Platelets were activated with 0.5 mM arachidonic acid (Sigma) for 10 minutes at 37°C and labelled with antibody CD61-PE (R&D systems) and PAC-1-FITC (BD) for GpIIbIIIa analysis. For PAC-1, platelets were fixed with paraformaldehyde as previously described [35]. Samples were acquired on a BD FACSCalibur cytometer. In order to avoid artefacts in fluorescence signal due to platelets' dimension and aggregation [36, 37], platelets were divided in two populations (R1 and R2) according to their FSC and CD61 staining. CD61 negative events, imputable to debris, were excluded from the analysis. R1 and R2 PAC-1 level has been expressed as mean fluorescence intensity.

For antioxidant evaluation, plasma in EDTA tubes (BD Vacutainer) was separated after centrifugation (at 1300 *g* at 48°C for 15 min) and stored at −80°C. Determination of sulfhydryl groups (SH) was performed using 5,50-dithiobis(2-nitrobenzoic acid) and the total radical-trapping antioxidant parameter (TRAP) and the FRAP were measured as previously described [31].

### 2.5. Statistical Analysis

Results showing a normal pattern were analysed by analysis of variance (ANOVA), others by Kruskal-Wallis one-way analysis of variance on ranks. The significance of the differences between treatments within the same time and those between the different times within the same treatment group were evaluated using the Student-Newman-Keuls method.

## 3. Results

### 3.1. Analysis of Vegetables

The TPC and the FRAP of the ANFI-Ex and W-Ex of the vegetables supplied to the subjects are shown in Table 2.

The higher average content of TPC and the greater FRAP was found in the W-Ex of the pepper (Table 2). On the other hand, although ANFI-Ex had lower TPC and FRAP, within them, red cabbage had the higher TPC and FRAP (Table 2).

### 3.2. Clinical, Immunological, and Antioxidant Markers

In Table 3 are described clinical parameters (glucose, insulin, TC, LDL, HDL, TG, BR, dir-BR, ind-BR, and proteins), markers of thrombotic risk (fibrinogen and D-dimer), platelet count, MPV, PDW, and ex vivo platelets' activation. No significant differences were found between all the markers analysed.

In particular, the analysis of the platelets' *ex vivo* stimulation with AA revealed that high vegetable consumption did not affect significantly PAC-1 expression. A nonsignificant decrease after high consumption of vegetables was observed for PAC-1 R2 AA (Table 3). Although this effect was accompanied by nonsignificant decreases in Pt counts and D-dimer concentrations, a trend of increase in fibrinogen has been observed and no differences were found in the *in vivo* markers of platelet activation MPV and PDW.

Dietary intervention did not affect red blood cell count (RBC 10^9/ml median (25%–75%): 4.6 (4.4–4.9) high T0, 4.7 (4.5–4.9) high T4; 4.7 (4.5–4.8) low T0, 4.6 (4.4–4.9) low T4), mean corpuscular volume (MCV fL median (25%–75%): 92.6 (88.5–94.4) high T0, 92.3 (88.5–94.5) high T4; 90.5 (85.4–92.9) low T0, 91.1 (85.4–92.9) low T4), and haemoglobin (Hb g/dl mean ± SD: 14.1 ± 1.3 high T0, 14.1 ± 1.4 high T4; 13.5 ± 1.2 low T0, 13.5 ± 1.4 low T4).

**Figure 2:**
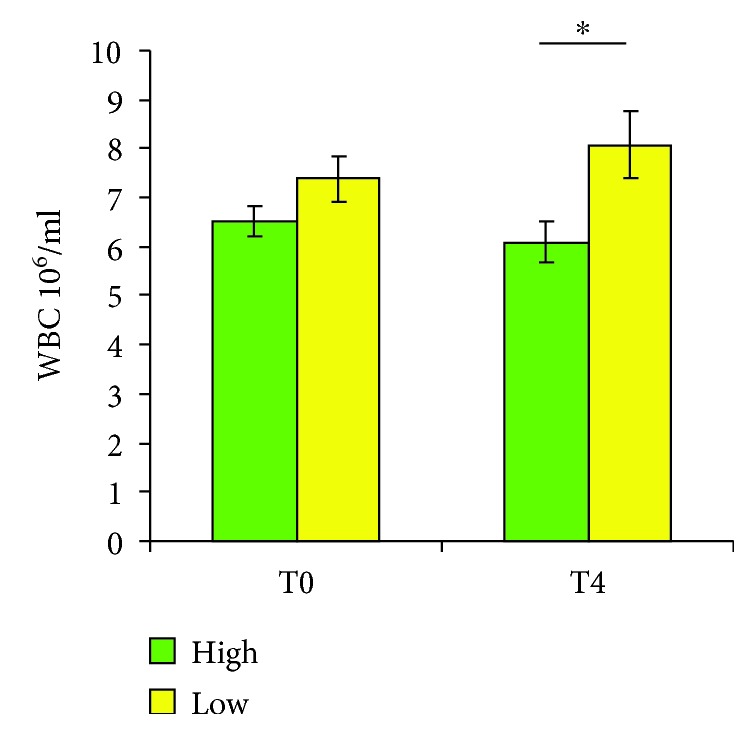
White blood cell count after interventions. Values at baseline (high T0 and low T0) and 4 weeks after high (4200 g/week) or low (800 g/week) vegetable consumption. Data are expressed as mean ± SEM (normality test passed; ANOVA applied, followed by Student-Newman-Keuls). High T4 versus low T4 ^∗^*p* < 0.05.

On the other hand, white blood cells (WBC) were significantly higher (*p* < 0.05) after 4 weeks of low intake of vegetables (low T4) compared to the high consumption (high T4) (Figure 2).

Moreover, due to the high variability within subjects, no significant differences were found in the counts of the different leukocyte populations (neutrophils 10^6/ml median (25%–75%): 3.8 (3.1–4.6) high T0, 3.9 (2.6–4.5) high T4; 4.0 (3.6–5.4) low T0, 4.3 (3.4–6.1) low T4; basophils 10^6/ml median (25%–75%): 0.0 (0–0.1) high T0, 0.0 (0–0.1) high T4; 0.0 (0–0.1) low T0, 0.0 (0–0.1) low T4; eosinophils 10^6/ml median (25%–75%): 0.2 (0.1–0.3) high T0, 0.2 (0.1–0.4) high T4, 0.2 (0.1–0.2) low T0, 0.20 (0.1–0.3) low T4; monocytes 10^6/ml mean ± SD: 0.50 ± 0.18 high T0, 0.49 ± 0.14 high T4; 0.50 ± 0.15 low T0, 0.54 ± 0.19 low T4; lymphocytes 10^6/ml median (25%–75%): 1.9 (1.4–2.3) high T0, 2.0 (1.50–2.2) high T4; 2.0 (1.5–2.4) low T0, 2.1 (1.9–2.7) low T4).

Low percentages (≤10%) of granulocytes, monocytes, and Treg expressed *β*-7 integrin (Table 4). The latter was present on about 20% of NK and CD4, with higher expression on CD4 naïve (28-29%), whereas percentages higher than 30 were found in B and CD8 populations (Table 4).

Neither lymphocyte subsets nor expression of *β*-7 integrin on leukocytes changed significantly after both low and high vegetable consumption (Table 4). Salivary IgA (IgAs) as well as plasma immunoglobulins and cytokines did not change significantly after both dietary interventions (Table 5). No significant differences in plasma and salivary antioxidants were observed (Table 6).

## 4. Discussion

In the present study, nonsignificant differences were found in antioxidant, inflammatory, and immune status, as well as in metabolic markers, after a 4-week high vegetable consumption.

Despite the small sample sizes could led to ineffective randomization and potential confounding, other crossover [38, 39] or larger [40, 41] trials, with high fruit and/or vegetable intakes [40] or high dietary total antioxidant capacity (TAC) diets [38, 39], did not observe significant effects on markers of glucose metabolism [38–41], TG [38, 39], TC [38, 39], FRAP [38, 39], leukocyte count [39], and inflammatory markers [40, 41]. Moreover, Valtueña et al. observed an unexpected decrease in plasma malondialdehyde after a diet with low TAC but not after the high-TAC diet [39]. This result reflects the difficulty of monitoring potential confounding during nutritional interventions in humans. In the present study, despite the exclusion of 5 subjects who reported symptoms of viral influence during the study, we cannot exclude nonreported events that could account of the observed increase in WBC count after low vegetable consumption.

Moreover, the evaluation of the effects of plant foods in humans presents many difficulties, including the healthy status of the selected subjects and the portion size of treatment and control groups. We used 3 servings of the portion size for vegetables (200 g) suggested by IV Revision of LARN (Intake Levels of Reference of Nutrients and energy for Italian population http://www.sinu.it/public/20141111_LARN_Porzioni.pdf), thus we compared 600 g/d (3 portions) versus 114 g/d of vegetables, including organic carrots, topinambur, tomatoes, red cabbage, and sweet peppers. Despite the high portion size of vegetables and the CVD risk factors of subjects in our study, we did not observe significant changes in any marker and our results are in line with that of Crane et al. [42]. In a crossover design overweight, postmenopausal women consumed for 3 weeks: 130 g, 287 g, and 614 g servings/d of fresh, greenhouse-grown vegetables, including baby carrots, baby leaf lettuce green mix, red bell peppers, and tomatoes [42]. Urinary 8-isoprostane F2*α* and serum high sensitivity C-reactive protein (CRP) were unchanged, despite the dose-response increase in plasma total carotenoids [42]. Moreover, in the study of Briviba et al. [4], vegetable intake (4.3 ± 0.6, 1 serving = 100 g, 430 g/d) included broccoli, brussels sprouts, cabbage, carrots, cauliflower, corn, cucumber, fennel, green beans, kohlrabi, lettuce, peas, radish, red cabbage, spinach, tomato, and zucchini. Authors did not observe differences in markers of DNA damage, lipid peroxidation (malondialdehyde and 8-iso-prostaglandin-F2alpha), and plasma trolox-equivalent antioxidant capacity, between 430 g/d and 100 g/d of vegetables [4]. However, Briviba et al. [4] evaluated the effect of the increase of both fruit and vegetable consumption (fruit 1 versus 3.5 servings/d, one serving = 100 g fruit or 200 ml juice).

In a recent meta-analysis [43], intervention studies with fruit and vegetable (including juices and extracts) intake decreased circulating levels of C-reactive protein and TNF-*α* but not IL-6. However, concerning the 8 trials that evaluated the anti-inflammatory effect of vegetable intake, only 3 observed decreases in CRP, TNF-*α*, and IL-6, two of which involve supplements (garlic or tomato extract) [43]. Moreover, the significant increase in the *γδ*-T cell population (mean difference: 1.68; *p* = 0.02), that have a role at the level of epithelial barriers (including bowel) [44], came from only 3 studies (93 treatments and 89 controls), of which 2 interventions with supplements (garlic extract and capsules of fruit and vegetable concentrate juice) and one with grape juice [43]. In our study, the gut-homing *β*7 integrin was not affected by high vegetable consumption. Therefore, no conclusive data are available on the effect of plant foods on gut-associated lymphocytes.

## 5. Conclusion

The study provides additional evidence about the uncertainty of providing a clear evidence for vegetables in modulating markers of immune function and antioxidant status. Further studies, including also the evaluation of potential changes in the human gut microbiota [45], are need in order to unravel the mechanism of effect of vegetable consumption in cardiovascular prevention.

## Figures and Tables

**Table 1:** Baseline characteristics and dietary intakes of included subjects.

	High (*n* = 15)	Low (*n* = 15)
BMI (kg/m^2^)	26.61 ± 4.09	24.56 ± 4.39
Smokers (*n*)	60% (9/15)	60% (9/15)
Dyslipidaemic subjects (*n*)	67% (10/15)	53% (8/15)
Kcal/day	1959 ± 239	1873 ± 702
Carbohydrates (g/day)	236 ± 51	218 ± 76
Protein (g/day)	70 ± 13	72 ± 27
Lipid (g/day)	75 ± 11	76 ± 27

High (4200 g/week) or low (800 g/week) vegetable consumption. BMI: body mass index. Data are expressed as mean ± SD.

**Table 2:** Antioxidant capacity and flavonoid content of vegetables provided for the study.

	W-Ex		ANFI-Ex	
	*FRAP μM*	*TPC μg GAE/g*	*FRAP μM*	*TPC μg GAE/g*
Carrot	0.00	11.46 ± 2.62	22.70 ± 27.10	12.41 ± 5.69
Red cabbage	250.52 ± 108.69	37.01 ± 22.81	361.85 ± 214.89	48.71 ± 14.73
Pepper	737.29 ± 261.93	59.05 ± 12.77	229.21 ± 76.01	26.36 ± 3.81
Tomato	110.15 ± 70.70	13.24 ± 1.41	0.00	8.32 ± 3.37
Topinambur	0.00	10.47 ± 0.91	0.00	13.85 ± 4.08

FRAP: ferric-reducing antioxidant power; TPC: total phenol content; GAE: gallic acid equivalents; ANFI-Ex: extracts containing the medium polar compounds; W-Ex: water extracts. The data are the mean ± SD of 4 different samples collected during the study analysed in triplicate.

**Table 3:** Clinical markers, thrombotic risk, and platelet activation.

	High T0	High T4	Low T0	Low T4
Glucose mg/dl	90.3 ± 5.5	94.0 ± 7.4	89.6 ± 4.3	90.6 ± 6.8
Insulin *μ*UI/ml	8.1 (5.4–11.8)	8.1 (5.0–9.4)	6.8 (5.0–9.4)	7.9 (5.3–10.5)
HOMA-IR	1.7 (1.1-2.6)	1.8 (1.1-2.1)	1.4 (1.0-1.9)	1.6 (1.1-2.3)
TC mg/dl	217.1 ± 35.8	217.3 ± 38.3	207.9 ± 41.0	204.8 ± 40.3
LDL mg/dl	140.1 ± 27.0	137.7 ± 28.9	127.9 ± 35.1	125.4 ± 33.7
HDL mg/dl	58.9 ± 8.1	60.5 ± 7.7	58.3 ± 8.5	57.3 ± 9.3
TG mg/dl	88 (65–104)	86 (73–100)	110 (64–161)	84 (69–176)
BR mg/dl	0.5 (0.4–0.6)	0.5 (0.4–0.7)	0.6 (0.3–0.7)	0.5 (0.3–0.6)
dir-BR mg/dl	0.12 (0.11–0.15)	0.12 (0.11–0.16)	0.14 (0.09–0.17)	0.13 (0.10–0.16)
ind-BR mg/dl	0.38 (0.29–0.49)	0.38 (0.31–0.54)	0.45 (0.21–0.55)	0.37 (0.23–0.47)
Proteins g/dl	6.8 ± 0.3	6.9 ± 0.1	6.8 ± 0.5	7.0 ± 0.4
Fibrinogen mg/dl	336.9 ± 39.7	340.9 ± 43.9	319.3 ± 43.3	325.2 ± 46.9
D-dimer ng/ml	323.7 (251.6–412.3)	288.5 (234.7–397.6)	277.3 (213.5–427.9)	320.2 (228.1–414.3)
Pt 10^3/microL	262 (223–288)	244 (212–293)	248 (219–301)	245 (220–336)
MPV fL	8.6 ± 0.9	8.6 ± 0.9	8.5 ± 0.7	8.4 ± 0.7
PDW %	16.3 ± 0.4	16.3 ± 0.4	16.3 ± 0.5	16.4 ± 0.5
PAC-1 R1	140.5 (49.1–226.3)	156.2 (135.7–187.8)	122.6 (98.5–255.1)	199.8 (151.1–291.1)
PAC-1 R1 AA	159.4 (48.8–174.4)	155.7 (147.1–212.7)	151.8 (62.9–193.2)	206.2 (129.9–256.0)
PAC-1 R2	234.2 (34.9–373.7)	138.2 (99.8–187.5)	277.5 (68.2–411.8)	260.1 (183.1–296.8)
PAC-1 R2 AA	213.8 ± 150.7	132.5 ± 78.3	215.6 ± 124.2	169.0 ± 98.9

Values at baseline (high T0 and low T0) and 4 weeks after high (4200 g/week) or low (800 g/week) vegetable consumption. Data are expressed as mean ± SD (normality test passed; ANOVA applied) or median (25%, 75%) (normality test failed; Kruskal-Wallis ANOVA on ranks applied). Pt: platelets; MPV: mean platelet volume; PDW: platelet distribution width; PAC-1 expression was measured after ex-vivo platelets' activation (AA: arachidonic acid; R1 and R2 gates are depicted in Figure 2); TC: total cholesterol; TG: triglycerides; UA: uric acid.

**Table 4:** Percentages of leukocytes and lymphocytes' subsets expressing *β*7 integrin.

%	High T0	High T4	Low T0	Low T4
Granulocytes *β*7+	0.8 (0.4–1.1)	0.7 (0.5–1.0)	0.6 (0.5–0.8)	0.7 (0.4–0.9)
Monocytes *β*7+	6.1 ± 2.1	5.9 ± 2.1	6.8 ± 1.9	6.5 ± 2.7
NK	7.8 (5.9–14.2)	10.2 (9.0–15.4)	8.2 (5.0–13.3)	10.9 (6.1–12.2)
NK *β*7+	18.3 ± 7.2	22.2 ± 5.6	19.4 ± 6.7	22.2 ± 6.9
B	6.8 (5.9–9.4)	8.8 (3.7–10.9)	9.8 (6.8–14.7)	8.3 (5.9–11.5)
B *β*7+	47.7 ± 11.5	48.9 ± 11.4	44.6 ± 17.9	47.6 ± 13.7
T	83.0 ± 5.3	79.4 ± 5.4	80.1 ± 5.3	80.8 ± 5.2
T *β*7+	30.4 ± 4.6	30.3 ± 6.7	31.1 ± 5.4	32.2 ± 7.0
CD8	26.7 (24.3–29.1)	27.0 (24.4–29.0	28.3 (24.4–33.3)	27.8 (22.5–34.5)
CD8 *β*7+	45.5 ± 9.0	45.5 ± 11.5	44.7 ± 10.2	45.5 ± 11.3
CD8 naïve	36.1 ± 12.0	35.7 ± 10.1	40.4 ± 15.9	39.6 ± 13.8
CD8 naïve *β*7+	46.4 ± 8.7	45.4 ± 10.3	45.0 ± 8.6	45.7 ± 8.5
CD8 memory	63.9 ± 12.0	64.3 ± 10.1	59.6 ± 15.9	60.3 ± 13.8
CD8 memory *β*7+	44.6 ± 9.9	45.4 ± 12.8	43.9 ± 11.4	45.5 ± 12.9
CD4	73.3 (70.9–75.7)	73.0 (71.0–75.6)	71.7 (66.6–75.6)	72.2 (65.5–77.5)
CD4 *β*7+	22.0 ± 3.7	21.7 ± 4.7	22.6 ± 4.8	23.3 ± 5.2
CD4 naïve	58.4 ± 12.4	55.4 ± 13.6	55.2 ± 10.0	52.7 ± 9.5
CD4 naïve *β*7+	28.4 ± 3.5	28.3 ± 5.7	28.7 ± 4.7	29.7 ± 6.7
CD4 memory	41.6 ± 12.4	44.6 ± 13.6	44.8 ± 10.0	47.3 ± 9.4
CD4 memory *β*7+	21.3 ± 5.5	21.7 ± 6.0	21.0 ± 6.6	23.0 ± 5.9
CD4CD25−	49.6 ± 7.3	49.2 ± 9.0	53.9 ± 8.6	52.0 ± 6.6
CD4CD25− *β*7+	21.6 ± 4.7	20.5 ± 5.1	21.2 ± 5.7	21.8 ± 5.7
CD4CD25dim	44.4 ± 6.3	45.3 ± 7.9	40.9 ± 7.8	43.0 ± 6.3
CD4CD25dim *β*7+	19.8 ± 4.0	19.8 ± 4.9	21.6 ± 5.2	22.6 ± 5.3
Treg	5.9 ± 1.3	5.5 ± 1.6	5.5 ± 0.7	5.1 ± 1.3
Treg*β*7+	10.9 ± 2.3	9.2 ± 3.4	10.5 ± 3.1	9.2 ± 2.8

Values at baseline (low T0 and high T0) and 4 weeks after low (800 g/week) or high (4200 g/week) vegetable consumption. Data are expressed as mean ± SD (normality test passed; ANOVA applied) or median (25%, 75%) (normality test failed; Kruskal-Wallis ANOVA on ranks applied).

**Table 5:** Immunoglobulins and cytokines.

	High T0	High T4	Low T0	Low T4
IgAs*μ*g/dl	1.0 ± 0.4	1.3 ± 0.7	0.6 ± 0.4	1.1 ± 0.7
IgA ml/dl	147.0 (125.0–203.0)	156.0 (118.0–203.0)	166.0 (118.7–204.2)	157.0 (121.5–185.0)
IgE IU/ml	45.5 (21.5–157.5)	48.5 (19.5–125.5)	18.0 (2.0–53.0)	26.0 (6.0–69.0)
IgG ml/dl	1049.1 ± 110.1	997.0 ± 133.4	1039.6 ± 170.5	1009.2 ± 170.0
IgM ml/dl	107.0 (65.0–155.0)	105.0 (73.0–163.0)	142.5 (74.0–192.7)	141.0 (70.7–186.5)
TGF-*β*pg/ml	1.2 ± 0.7	1.3 ± 0.7	1.3 ± 0.6	1.3 ± 0.5
IL-17 pg/ml	2.4 (0.3–3.5)	2.7 (0.6–3.0)	0.8 (0.4–2.5)	1.6 (0.5–2.2)
TNF-*α*pg/ml	8.3 (8.2–9.0)	8.3 (7.9–8.5)	8.6 (8.2–9.0)	8.6 (8.1–9.0)
IL-6 pg/ml	7.9 (7.7-8.8)	8.5 (7.7–9.2)	8.8 (8.0–9.6)	8.4 (7.9–9.4)
IL-10 pg/ml	8.3 (8.1–9.0)	8.7 (8.3–9.1)	8.7 (8.3–9.2)	8.7 (8.4–8.9)
IL-4 pg/ml	7.4 (7.2–7.6)	7.5 (7.1–8.4)	7.9 (7.3–8.1)	7.7 (7.5–8.2)
IL-2 pg/ml	7.0 (6.9–7.6)	7.4 (7.1–7.7)	7.7 (7.3–7.9)	7.6 (7.4–7.7)
IFN-*γ*pg/ml	8.1 ± 0.5	8.1 ± 0.5	8.3 ± 0.7	8.2 ± 0.5

Values at baseline (high T0 and low T0) and 4 weeks after high (4200 g/week) or low (800 g/week) vegetable consumption. Data are expressed as mean ± SD (normality test passed; ANOVA applied) or median (25%, 75%) (normality test failed; Kruskal-Wallis ANOVA on ranks applied). IL: interleukin; INF: interferon; Ig: immunoglobulins; salivary IgA: IgAs; TGF: transforming growth factor; TNF: tumor necrosis factor.

**Table 6:** Plasma and salivary antioxidants.

	High T0	High T4	Low T0	Low T4
TRAP *μ*M (plasma)	902.7 (866.5–1066.1)	929.7 (831.1–1103.6)	945.3 (857.3–1086.8)	944.4 (850.5–1135.8)
SH *μ*M (plasma)	586.8 ± 69.4	555.6 ± 78.4	586.8 ± 92.1	547.5 ± 94.6
UA *μ*M (plasma)	190.4 (154.7–243.9)	196.3 (136.8–238.0)	214.2 (166.6–238.0)	202.3 (178.5–226.1)
FRAP *μ*M (plasma)	666.2 (568.2–693.0)	688.6 (561.8–790.2)	673.1 (581.5–799.6)	635.8 (585.4–720.1)
TRAP *μ*M (saliva)	432.2 ± 129.8	374.7 ± 112.7	380.0 ± 114.0	389.1 ± 114.3
FRAP *μ*M (saliva)	498.9 (368.6–611.8)	451.7 (371.7–596.9)	563.1 (407.3–665.1)	474.6 (278.1–578.6)
UA *μ*M (saliva)	127.9 ± 13.7	126.7 ± 14.1	131.5 ± 17.5	128.5 ± 16.5

Values at baseline (high T0 and low T0) and 4 weeks after high (4200 g/week) or low (800 g/week) vegetable consumption. Data are expressed as mean ± SD (normality test passed; ANOVA applied) or median (25%, 75%) (normality test failed; Kruskal-Wallis ANOVA on ranks applied). TRAP: radical-trapping antioxidant parameter; FRAP: ferric-reducing antioxidant power; SH: sulfhydryl groups; UA: uric acid.

## Data Availability

The data used to support the findings of this study are restricted by the San Raffaele Ethics Committee in order to protect patient privacy. Data are available from Mauro Serafini for researchers who meet the criteria for access to confidential data.
